# Improved Antioxidant, Antihypertensive, and Antidiabetic Activities and Tailored Emulsion Stability and Foaming Properties of Mixture of Corn Gluten and Soy Protein Hydrolysates Via Enzymatic Processing and Fractionation

**DOI:** 10.1002/fsn3.4532

**Published:** 2024-10-23

**Authors:** Homaira Mirzaee, Hassan Ahmadi Gavlighi, Mehdi Nikoo, Chibuike C. Udenigwe, Amir Rezvankhah, Faramarz Khodaiyan

**Affiliations:** ^1^ Department of Food Science and Technology, Faculty of Agriculture Tarbiat Modares University Tehran Iran; ^2^ Halal Research Center of IRI, Iran Food and Drug Administration Ministry of Health and Medical Education Tehran Iran; ^3^ Department of Pathobiology and Quality Control, Artemia and Aquaculture Research Institute Urmia University Urmia Iran; ^4^ School of Nutrition Sciences University of Ottawa Ottawa Ontario Canada; ^5^ Department of Food Science and Technology, Razi Food Chemistry Lab, College of Agriculture and Natural Resources University of Tehran Karaj Iran; ^6^ Bioprocessing and Biodetection Laboratory, Department of Food Science and Engineering University of Tehran Karaj Iran

**Keywords:** α‐amylase inhibition, emulsion stability, hydrophobicity, mixed soy and corn hydrolysates, ultrafiltration

## Abstract

Bioactive peptides and protein hydrolysates have gained considerable attention in the food industry and functional food markets due to their diverse health effects, including antioxidant, antihypertensive, and antidiabetic properties. This study aimed to produce combined soy and corn protein hydrolysates using Alcalase (Al), modification of Al‐hydrolysates through sequential hydrolysis using Flavourzyme (Al‐FL), cross‐linking of Al‐hydrolysates using microbial transglutaminase (MTGase) (Al‐TG), and fractionation of Al‐hydrolysates by ultrafiltration (UF) with molecular weight (MW) cut‐off of 100 (Al‐F4), 30 (Al‐F3), 10 (Al‐F2), and 2 kDa (Al‐F1). Notably, the < 2 kDa fraction (Al‐F1) showcased exceptional biological activities, including antioxidant (81.54% DPPH, 98.02% ABTS), antihypertensive (95.45%), and antidiabetic effects (44.72% α‐glucosidase, 77.52% α‐amylase), linked to its high hydrophobic amino acid content and low molecular weights (111 and 263 Da). Conversely, the higher molecular weight fraction (Al‐TG) excelled in emulsion and foam stability, attributed to its balanced amino acid profile and larger peptides (1385–7057 Da). Our findings reveal that specific protein hydrolysate fractions, particularly Al‐F1 and Al‐TG, are promising for applications in food and pharmaceutical formulations due to their enhanced biological and functional properties.

## Graphical abstract

Formulated mixed corn and soy peptides modified by enzyme and ultrafiltration. Enzymatic cross‐linking hydrolysates had higher emulsion and foam properties. The smaller fraction of hydrolysate showed higher biological activities.

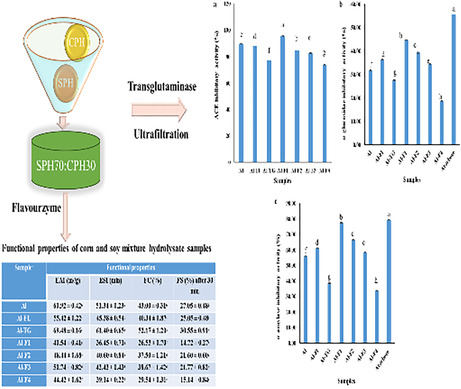

## 1 Introduction

Bioactive peptides and protein hydrolysates are increasingly recognized in the food industry and functional food markets for their various health benefits, including antioxidant activity, blood pressure management, and blood glucose regulation (Yu et al. 2023). Especially, plant‐based proteins have widely attracted attention to develop peptides with high potential in biological activities (Phongthai et al. 2018). The biologically active amino acids of corn peptides are alanine, valine, leucine, isoleucine, methionine, proline, and phenylalanine as hydrophobic amino acids, while histidine, tyrosine, and lysine as hydrophilic amino acids have potent biological activities (Liu, Fang, et al. 2020). Also, the biological active amino acids of soy peptides are lysine, proline, isoleucine, leucine, valine, and alanine (Zhang et al. 2021). The most preferable and controllable method to generate bioactive peptides is enzymatic hydrolysis of proteins (Dabbour et al. 2020). It involves controlling the degree of hydrolysis (DH), and then isolation or purification of produced peptides using membrane systems (Zhou, Sun, and Canning 2012). Moreover, it has been shown that the formation of cross‐links between produced peptides has led to enhanced antidiabetic, antihypertensive, and functional activities (Wang et al. 2016). There are several proteases with extensive applications in the development of the peptides with improved antiradical activity, hypertension regulation, and blood glucose control (Fadimu, Farahnaky, et al. 2022; Fadimu et al. 2021; Fadimu, Gill, et al. 2022; Liu, Song, et al. 2020; Rahimi et al. 2022; Rezvankhah et al. 2021a). Alcalase and Flavourzyme are widely used commercial enzymes for generating peptides from plant‐based proteins with improved nutritional and health‐promoting attributes (de Matos et al. 2022). Alcalase cleaves peptides internally, releasing sequences with hydrophobic patches that are strongly involved in antioxidant, antihypertensive, and antidiabetic activities (Fadimu, Farahnaky, et al. 2022; Fadimu, Gill, et al. 2022). In contrast, Flavourzyme cleaves proteins from external and internal sites, producing small peptides and free amino acids that are mostly involved in taste development and are known as a sweetening enzyme (Xu et al. 2020). Two‐stage hydrolysis of corn and soy proteins using Alcalase and Flavourzyme can also be investigated to assess the biological activities and functional properties of produced peptides. Sequential hydrolysis affects peptide sequences, surface properties, and ultimately biological activities. Using Flavourzyme as a second enzyme reduces bitter peptides generated initially, mainly by decreasing hydrophobic patches. Factors such as degree of hydrolysis, peptide sequences, amino acid profiles, and molecular weight (MW) are important for biological activities. Sequential hydrolysis can also alter the accessibility of active peptides, either by increasing or decreasing them (Zeng et al. 2023).

Microbial transglutaminase (MTGase) has been used to cross‐link proteins and peptides with the aim of improving functional properties (Liu et al. 2022). Moreover, MTGase has been introduced to soy protein hydrolysates (SPH) to promote umami taste by increasing the amount of peptides with MW of 2–5 kDa (Song et al. 2013). Liu et al. (2022) prepared corn glutelin hydrolysates using Alcalase and then glycosylated them with glucosamine via MTGase to modify the main and side chains. The glycosylated hydrolysates exhibited improved solubility across pH 2–11 and enhanced functional properties—except for foaming stability—compared to glutelin. Additionally, they showed significantly improved digestibility and strong antioxidant activities. The main mechanism for improving functional properties involves designing new peptides with a well‐balanced hydrophobic/hydrophilic amino acid composition (Shi et al. 2018). Fractionation of peptides obtained from enzymatic hydrolysis has been used as an efficient method to separate the most promising peptides (Yu et al. 2023). Ultrafiltration (UF) as a membrane method can be considered the most common method of fractionating protein hydrolysates into peptides with various MWs. Fractionated peptides facilitate the identification of peptides with high potential in antioxidant, antihypertensive, and antidiabetic activities (Hu et al. 2022). For example, peptides with MW < 1 and 2–5 kDa have high hydrophobicity and potentially can interact with free radicals and inhibit the enzymes relevant to the treatment of hypertension and diabetes (Aondona et al. 2021). Jin et al. (2015) also hydrolyzed corn gluten with various enzymes, including Alcalase and Flavourzyme combinations. The hydrolysate produced with Alcalase–Flavourzyme showed superior antioxidant activities. Upon purification, the peptide containing cysteine displayed strong reducing power and impressive abilities to scavenge both DPPH radical and superoxide anion radical.

Mixed and modified protein hydrolysates from corn and soy peptides were developed in our previous research using Alcalase (Mirzaee et al. 2023). However, the consecutive modifications of mixed soy and corn peptides for improved functionality and bioactivity have not been studied. Hence, the objective of this study was to hydrolyze, cross‐link, and fractionate the mixed soy and corn‐derived peptides through the sequential hydrolysis process using Flavourzyme, MTGase‐mediated cross‐linking, and membrane separation by UF. The effects of each process were investigated on amino acid composition, hydrophobicity, MW, in vitro antioxidant, antihypertensive and antidiabetic activities, and functional features.

## 2 Materials and Methods

### 2.1 Materials

Two proteolytic enzymes, including 2.4 L of Alcalase and 1000 L of Flavourzyme were purchased from Novozymes Co. (Bagsværd outside of Copenhagen). Diabetes‐related enzymes including α‐amylase (porcine pancreatic) and α‐glucosidase (intestinal acetone powder from rat), 4‐nitrophenyl *α*‐d‐glucopyranoside (PNPG), Angiotensin‐converting enzyme (ACE), N‐Hippuryl‐L‐histidyl‐L‐leucine hydrate, and 1‐anilino‐8‐naphthalene‐sulfonic acid (ANS) were provided from Sigma‐Aldrich (St, Louis, MO, USA). All chemicals used were of analytical and HPLC grade.

### 2.2 Processing and Modification Methods

#### 2.2.1 Fractionation

The combined hydrolysate of soy protein and corn protein (70:30, w/w) was prepared using the method outlined in our prior study (Mirzaee et al. 2023). Briefly, soy protein isolate (SPI) and corn gluten meal (CGM) were separately hydrolyzed using Alcalase (2.5% w/w). After deactivation, the enzyme and separation of unhydrolyzed peptides, soy protein hydrolysate (SPH), and corn protein hydrolysate (CPH) were mixed together at a ratio of 70:30 based on the obtained results regarding the highest inhibition against the ACE, α‐glucosidase, and α‐amylase. Then, fractionation of the hydrolysate was conducted using an UF system (Sartorius, viva flow 200) with cross‐flow state which had MW cut‐offs of 100, 30, 10, and 2 kDa. The process involved filtering the mixed soy protein hydrolysate (70% w/w) and corn protein hydrolysate (30% w/w), which was developed in our previous study using Alcalase and termed SPH70:CPH30, through the respective membranes. The resulting fractions were labeled as Al‐F1 (MW < 2 kDa), Al‐F2 (MW 2–10 kDa), Al‐F3 (MW 10–30 kDa), and Al‐F4 (MW 30–100 kDa). Subsequently, the produced samples were converted into powders using a freeze‐drying system, then stored at −18°C until analysis.

#### 2.2.2 Enzymatic Processing

##### 2.2.2.1 Sequential Hydrolysis

SPH70:CPH30, obtained by Alcalase (mentioned in Section 2.2.1), with a degree of hydrolysis (DH) of 15%, was subjected to further hydrolysis using Flavourzyme. The sequential hydrolysis of the hydrolysate was performed using a Flavourzyme concentration of 5% (w/w) at an optimum temperature of 50°C, pH 7.0, and a duration of 45 min. The hydrolysis process resulted in a final DH of 20%. The DH was determined based on the pretreatments applied. To terminate the enzyme activity, the peptide solution was heated at 95°C for 15 min after the hydrolysis process. It was then centrifuged at 15,000 × *g* for 20 min to separate the hydrolyzed peptides. The resulting supernatant was subsequently converted into powder using a lab‐scale spray dryer (Buchi 290 unit, Switzerland), while the inlet temperature was set at 165°C and the outlet temperature was controlled at 80°C to 85°C. Blowing air had a pressure of 0.2–0.3 MPa. The powdered peptide product obtained was labeled as Al‐FL and stored at −18°C for analysis.

##### 2.2.2.2 MTGase‐Mediated Cross‐Linking

SPH70:CPH30 (10%, w/w) was subjected to cross‐linking using MTGase, following the method described previously (Rezvankhah, Yarmand, and Ghanbarzadeh 2022). In summary, MTGase was used at a concentration of 1.2% (w/w), with the enzyme temperature set at the optimum of 45°C, pH at 8.0, and a reaction time of 5 h. Afterward, the enzyme activity was halted by heating the treated peptide solution to 90°C for 15 min. The solution was cooled to 37°C, and then, centrifuged at 11,300 × *g* for 10 min at 25°C to separate the supernatant rich in cross‐linked peptides. To make the supernatant dried, spray dryer was used. The resulting product, consisting of cross‐linked peptides, was labeled as Al‐TG and kept under the freezer condition.

### 2.3 Amino Acid Profile

The amino acid composition of the modified samples was assessed using reverse‐phase high‐performance liquid chromatography (RP‐HPLC) employing an Agilent 1100 HPLC system from Agilent Ltd. The system had an analytical C_18_ column (diameter and length of 4 × 250 mm with particle size of 5 μm; Zorbax, Agilent), plugged to a fluorescence detector (excitation: 330 nm, emission: 480 nm, LC305 Lab Alliance). The chromatographic separation of the amino acids was performed at a flow rate of 1 mL/min and a separation temperature of 35°C (Nikoo et al. 2019).

### 2.4 Surface Hydrophobicity ( *H*_0_ )

The surface hydrophobicity of the modified samples was assessed at various concentrations (0.01–0.02 mg/mL) in 10 mM phosphate buffer (pH 7.0), following the method described by Mirzaee et al. (2023). Four milliliters of sample solutions were combined with 50 μL of 9 mmol/L ANS in 0.2 mol/L phosphate buffer (pH 7.0) to evaluate surface hydrophobicity. The plots that depicted the relative fluorescence intensity versus soluble protein content were used to estimate the initial slopes (*H*_0_) by employing a linear regression, serving as indicators of surface hydrophobicity.

### 2.5 Molecular Weight Profile

The MW profile of the modified samples was determined using a horizontal sodium dodecyl‐sulfate polyacrylamide gel electrophoresis (SDS‐PAGE) according to the method of Rezvankhah et al. (2024) using a buffer containing 1.2% of SDS, 0.30% of 2‐ME, 0.02% of bromophenol blue, and 20% of pH 7.5 tris buffer adjusted with acetic acid. Samples were analyzed in a horizontal electrophoresis unit at a voltage of 160 V. For this aim, samples were loaded into 12% precast gels (Mini‐Protean TGX, Bio‐Rad, CA, USA). The gels were stained for 3 h using an aqueous‐methanolic solution consisting of 45% (v/v) methanol, 10% (v/v) acetic acid, and 45% (v/v) deionized water with 120 mg/mL of Coomassie Brilliant Blue‐R‐250. Subsequently, the samples were destained using a mixture of 45% water, 45% methanol, and 10% acetic acid. To determine MW of the polypeptide bands in the hydrolysate samples, commercial standard protein markers (Bio‐Rad Broad Range Marker) were used.

### 2.6 MW of Peptides

The MW distribution of peptides was measured using size‐exclusion high‐performance liquid chromatography (Waters 600 HPLC, Waters Co., Milford, MA, USA) coupled with a TSK gel 2000 SWXL (300 × 7.8 mm) column (Tosoh, Tokyo, Japan) (Karimi et al. 2020). Cytochrome C (12,384 Da), bacitracin (1422 Da), Gly‐Gly‐Tyr‐Arg (451 Da), and Gly‐Gly‐Gly (189 Da) were used as the molecular weight standards.

### 2.7 Antioxidant Activity

#### 2.7.1 DPPH ^·^ Scavenging Assay

The DPPH^·^ radical scavenging activity (RSA) of the modified samples was assessed using a modified method as suggested by Rezvankhah et al. (2021b). Briefly, 500 μL of sample solutions (10 mg/mL) were mixed with 500 μL of 2,2 diphenyl‐1‐picrylhydrazyl solution (0.2 mM). Then, the obtained mixture was incubated at a dark place for 40 min. The absorbance of sample solutions was determined at 517 nm using a UV‐spectrophotometer. Ascorbic acid was considered to be compared with hydrolysate samples (positive control). The RSA (%) was computed using the equation below:

(1) $$\mathrm{RSA}\left(\%\right)=\frac{A_{C}-A_{S}}{A_{C}-A_{B}}\times 100$$

where *A*_C_, *A*_S_, and *A*_B_ indicate the absorbance of control, sample, and blank, respectively.

#### 2.7.2 ABTS^·^ ^+^ Scavenging Assay

The ABTS^·+^ radical scavenging activity (RSA) of the modified samples was assessed following the method reported by Rahimi et al. (2022) with some modifications. Briefly, 60 μL of sample solutions (10 mg/mL) were mixed with 960 μL of diluted ABTS^·+^ solution. The mixtures were stood for 10 min at the ambient temperature in the dark, and the absorbance was measured at 734 nm. Ascorbic acid was used as positive control. The ABTS RSA (%) was determined using Equation 1.

### 2.8 In Vitro Antihypertensive Activity

The ACE‐inhibitory activity of the modified samples was determined using the method reported by Mirzaee et al. (2023). ACE inhibition was determined using N‐Hippuryl‐L‐histidyl‐L‐leucine hydrate (HHL). The method measures the amount of hippuric acid released from HHL after incubation with angiotensin I‐converting enzyme for 35 min at 37°C, comparing results with the sample present (peptides) and without it (control). The inhibition against the ACE was then computed using the following formula:

(2) $$\text{Inhibitory activity}\;\left(\%\right)=\left[\frac{\left(A_{c}-A_{s}\right)}{\left(A_{c}-A_{b}\right)}\right]\times 100$$

*A*_*c*_ exhibits the absorbance of the control, *A*_*s*_ relates to the absorbance of the sample, and *A*_*b*_ shows the absorbance of the blank.

### 2.9 In Vitro Antidiabetic Activities

#### 2.9.1 α‐Glucosidase Inhibition Assay

The inhibition against the α‐glucosidase was evaluated using a method adapted from Rahimi et al. (2022). Sample solutions were mixed with α‐glucosidase (diluted rat intestinal acetone powder), and the mixture was incubated at 37°C for 6 min. Then, PNPG solution was incorporated, and another 20‐min incubation at 37°C was followed, during which absorbance at 405 nm was measured every 2 min. A control was performed using sodium phosphate buffer instead of the samples, and acarbose (0.5 mg/mL) was used as a positive control. The inhibitory activity percentage of the samples was computed as follows:

(3) $$\text{Inhibition of}\;\alpha ‐\text{glucosidase}\;\left(\%\right)=\frac{\text{Absorbance}\;\left(\text{control}\right)-\text{Absorbance}\;\left(\text{sample}\right)}{\text{Absorbance}\;\left(\text{control}\right)}\times 100$$

#### 2.9.2 Porcine Pancreatic α‐Amylase Inhibition Assay

The inhibition of α‐amylase by the mixed corn and soy protein hydrolysates was evaluated using a modified version of the procedure detailed by Rahimi et al. (2022). In brief, 120 μL of sample solutions (0.5 mg/mL) were mixed with 120 μL of α‐amylase solution (0.6 U/mL) and incubated for 5 min at 37°C. After the initial reaction, the mixture was enhanced by adding 120 μL of a starch solution (0.5 g/100 mL) and incubated for an additional 20 min at the same temperature. To stop the reaction, the mixture was heated to 100°C for 10 min and then centrifuged at 13,000 × *g* for 3 min. Subsequently, 20 μL of the diluted supernatant was combined with 1 mL of PAHBAH and heated at 70°C for 10 min. The absorbance was then measured at 410 nm. Acarbose (0.5 mg/mL) served as the positive control in the experiment.
(4)

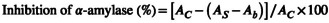


*A*_*C*_, *A*_*S*_, and *A*_*b*_ indicate control containing buffer, starch, and enzyme, sample containing peptides, starch, and enzyme, and background samples containing sample, starch, and buffer, respectively.

### 2.10 Functional Properties

#### 2.10.1 Solubility

The solubility of the hydrolysate samples was ascertained following the method outlined by Daliri et al. (2021) with slight modifications. In brief, 300 mg of each sample was dissolved in 20 mL of distilled water, and the pH was adjusted to 4.0, 7.0, and 9.0 using NaOH (1 N) and HCl (1 N). The mixture was heated to 35°C for 35 min while being stirred at 200 rpm. Afterward, the mixture was centrifuged for 15 min at 15,000 × *g*. The protein content of both the supernatant and the sample was determined using the Bradford (Bradford 1976) technique. Finally, the solubility was calculated as follows:

(5) $$\text{Solubility}\;\left(\%\right)=\frac{\text{The amount of protein in the supernatant}}{\text{The amount of protein in the sample}}\times 100$$

#### 2.10.2 Emulsion Activity Index (EAI) and Emulsion Stability Index (ESI)

The EAI and ESI of hydrolysate samples were determined according to Rezvankhah et al. (2024). Approximately 300 mg of the samples were dissolved in 30 mL of distilled water, with the pH adjusted to 7.0. Subsequently, approximately 10 mL of corn oil was introduced to the sample mixture and homogenized using an IKA homogenizer (Staufen, Germany) at a speed of 20,000 rpm for 1 min. immediately after homogenization, a 50 μL aliquot of the emulsion was taken, and another 10 min interval aliquot was taken after homogenization. A spectrophotometer with a wavelength of 500 nm was used to measure the absorbance of the diluted solution, and EAI and ESI were computed using the following equations:

(6) $$\mathrm{EAI}\;\left(m^{2}g^{‐1}\right)\left(\%\right)=\frac{2\times 2.303\times A_{0}}{0.25\times \text{protein weight}\;\left(g\right)}\times 100$$

(7) $$\mathrm{ESI}\;\left(\min\right)\left(\%\right)=\frac{A_{0}\times ∆t}{A_{0}-A_{10}}\times 100$$

*A*_0_ and *A*_10_ express the absorption of homogenized samples at a time of 0 min and a time of 10 min after homogenization. ∆t indicates the time interval (10 min).

#### 2.10.3 Foaming Capacity (FC) and Foaming Stability (FS)

The FC and FS of the hydrolysates were assessed using the method described by Rezvankhah et al. (2024). For the test, 300 mg of the sample was mixed with 25 mL of distilled water, and the pH of the solution was adjusted to 7.0. Then, the sample solutions were aerated at 20,000 rpm for 2 min using an IKA homogenizer (Staufen, Germany). The aerated peptide solutions were poured into a 100 mL graded cylinder and the total volume was recorded. The samples were also kept for 30 min at room temperature and then the volumes were determined. The FC and FS were calculated according to the equations provided below:

(8) $$\mathrm{FC}\;\left(\%\right)=\frac{B-A}{A}\times 100$$

(9) $$\mathrm{FS}\;\left(\%\right)=\frac{C-A}{A}\times 100$$

*A* represents the initial volume (in mL) before aeration (whipping); *B* denotes the foam volume (in mL) immediately after whipping (0 min), and *C* signifies the foam volume (in mL) after 30 min of whipping.

### 2.11 Fourier Transform Infrared (FTIR) Spectroscopy

The structure of the modified samples was investigated by Fourier transform infrared spectroscopy (FTIR). Briefly, the powders were mixed with KBr powder in a ratio of 1:10 and then pressed into a disk before spectrum acquisition. The spectrum was scanned in transmission mode from 450 to 4000 cm^−1^ (Rezvankhah et al. 2021a). Omnic 8.0 software (Thermo Fisher Scientific Inc., Madison, WI) and Peakfit 4.12 software (Systat Software, San Jose, CA) were also used to evaluate the data. The spectrum was coordinated and transformed using Omnic 8.0 software, and the spectral data was processed using Peakfit 4.12 to create Fourier self‐deconvolution and the second derivative of amide I at 1700–1600 cm^−1^ (Zhang et al. 2022).

### 2.12 Statistical Analysis

The results were presented as the mean standard deviation for all experiments that were carried out in triplicate. A one‐way analysis of variance (ANOVA) was performed using SPSS version 22 software to analyze the data. Significance differences between samples in each analysis were assessed using the Duncan test at a confidence level of 95% (*p* < 0.05).

## 3 Results and Discussion

### 3.1 Amino Acid Composition

The amino acid profiles of the hydrolysates are presented in Table 1. The subsequent processing of Al‐hydrolysates (SPH70:CPH30) through membrane ultrafiltration and enzymatic methods (using Flavourzyme and MTGase) resulted in the modification of the amino acid content. Among the fractions, Al‐F2 exhibited the highest hydrophilic amino acid content (51.24 g/100 g), whereas the lowest content (34.89 g/100 g) was observed in Al‐F1 (UF fraction). Two‐stage hydrolysis of SPH70:CPH30 (Al) resulted in a minor reduction in hydrophilic amino acid content (45.47 g/100 g for Al‐FL), while MTGase‐mediated cross‐linking significantly decreased the hydrophilic amino acid content (39.95 g/100 g for Al‐TG). Regarding the hydrophobic amino acids, the highest value (28 g/100 g) was obtained for Al‐F1 and the lowest value (17.06 g/100 g) was obtained for Al‐F4. The second hydrolysis by Flavourzyme and also cross‐linking using MTGase led to decrease in the hydrophobic amino acid contents (22.02 g/100 g for Al‐FL and 19.87 g/100 g for Al‐TG). The processing also changed the total amino acid contents. Second hydrolysis and cross‐linking of Al‐hydrolysates resulted in a decrease in total amino acid contents, with the highest effect observed after MTGase treatment. Our findings on the changes in amino acids were consistent with the results of previous research (Fadimu, Farahnaky, et al. 2022; Fadimu et al. 2021; Fadimu, Gill, et al. 2022). Indeed, after hydrolysis and cross‐linking processes, the unprocessed peptides are separated through centrifugation which can be considered the main influencing factor on amino acid composition. However, fractionation by passing the Al‐hydrolysates through the different membrane filters had different effects. Al‐F1 (UF fraction) and Al‐F4 demonstrated lower total amino acid contents, whereas Al‐F2 and Al‐F3 exhibited higher values. The fraction Al‐F2 had the highest content of bitterness‐contributing amino acids (30.32 g/100 g), while the lowest content (19.69 g/100 g) was observed in Al‐F4. Second hydrolysis and cross‐linking also resulted in a reduction of bitterness‐contributing amino acids. The most significant umami amino acid contents (27.49 and 27.48 g/100 g) were observed in Al‐F2 and Al‐F3, respectively, while the lowest content (16.48 g/100 g) was found in Al‐F1. The highest content of sweet amino acids (15.98 g/100 g) was identified in Al‐F2, whereas the lowest (9.76 g/100 g) was observed in Al‐F4. Regarding aromatic amino acids, Al‐F1 exhibited the highest content (10.49 g/100 g), while the lowest content (5.62 g/100 g) was observed in Al‐F4. As expected, the processes of hydrolysis, cross‐linking, and fractionation affected the MW of the generated peptides. Consequently, the peptide fragments displayed varying amino acid compositions (Aondona et al. 2021). Reduction of hydrophilic and hydrophobic amino acids in Al‐FL and Al‐TG could be associated with the separation of nonhydrolyzed and nonpolymerized peptides by centrifugation while the increase in hydrophilic and hydrophobic amino acids of Al‐F2, Al‐F3, and Al‐F1 could be due to the concentration of peptides with the specific amino acids in the fractions, as reported previously (Fadimu, Gill, et al. 2022). The higher hydrophobic amino acids in Al‐F1 and Al‐F2 fractions was in accordance with previous reports that several peptide fractions with low MWs had high hydrophobic amino acid contents (Karimi, Azizi, and Ahmadi Gavlighi 2020; Karimi et al. 2020). The amino acid compositions are crucial in determining the physicochemical and bioactive properties of peptides. For example, umami amino acid contents have been reported to be high in peptide fragments with MW of 2–5 and 2–10 kDa (Liu et al. 2012; Song et al. 2013). The peptides with high hydrophobic amino acids have been reported to have high bitterness (Liu et al. 2022). High content of aromatic amino acids (Tyr, Trp, Phe, and His) in fractions of Al‐F1 and Al‐F2 could support stronger antioxidant activity (Singh et al. 2022).

**TABLE 1:** Amino acid composition of corn and soy mixture hydrolysate samples.

Amino acid composition (g/100 g)	Hydrolysate samples^a^						
	SPH70:CPH30 (Al)	Al‐FL	Al‐TG	Al‐F1	Al‐F2	Al‐F3	Al‐F4
Hydrophilic							
Aspartic acid (Asp)	7.76	7.80	6.91	4.71	9.35	9.47	5.76
Glutamic acid (Glu)	16.78	16.49	14.84	11.77	18.14	18.01	13.19
Serine (Ser)	4.2	4.86	3.56	4.2	4.75	3.97	2.89
Glycine (Gly)	2.99	2.83	2.57	2.32	3.25	3.18	2.32
Histidine (His)	1.49	1.28	1.2	1.18	1.37	1.53	1.10
Arginine (Arg)	3.85	3.85	3.39	2.27	3.67	5.04	3.57
Threonine (Thr)	2.59	2.43	2.24	2.51	3	2.56	1.87
Tyrosine (Tyr)	2.90	2.77	2.43	4.19	3.47	2.68	1.82
Lysine (Lys)	3.34	3.16	2.81	1.74	4.24	3.0	3.40
Total hydrophilic amino acids	45.90	45.47	39.95	34.89	51.24	49.44	35.92
Hydrophobic							
Alanine (Ala)	4.29	4.22	3.63	5.71	4.98	3.54	2.68
Valine (Val)	3.13	3.02	2.74	2.93	3.79	3.16	2.22
Methionine (Met)	1.2	1.08	1	1.49	1.31	1.11	0.93
Isoleucine (Ile)	3.07	3.03	2.68	2.48	3.74	3.20	2.32
Leucine (Leu)	7.19	6.68	6.18	9.66	8.59	6.12	5.03
Phenylalanine (Phe)	3.74	3.52	3.23	5.12	4.38	3.27	2.70
Tryptophan (Try)	0.46	0.47	0.41	0.61	0.52	0.43	1.18
Total hydrophobic amino acids	23.08	22.02	19.87	28	27.31	20.83	17.06
Total amino acids (%w/w)	68.98	67.49	59.82	62.89	78.55	70.27	52.98
Bitterness	26.57	25.23	22.85	29.32	30.32	26.11	19.69
Umami	24.54	24.29	21.75	16.48	27.49	27.48	18.95
Sweetness	14.07	14.34	12	14.74	15.98	13.25	9.76
Aromatic amino acids	8.13	7.57	6.86	10.49	9.22	7.78	5.62

^a^ Al‐hydrolysate was obtained through the mixing of soy protein hydrolysate (SPH:70) and corn protein hydrolysate (CPH:30). Al‐Fl was sequential hydrolysis of SPH70:CPH30 using Flavourzyme. Al‐TG was sequential cross‐linking of SPH70:CPH30. Al‐F1, Al‐F2, Al‐F3, and Al‐F4 were fractionated hydrolysates obtained from Al‐hydrolysate, with MW of < 2, 2–10, 10–30, and 30–100 kDa, respectively. Data are means ± SD of three replicates.

### 3.2 Surface Hydrophobicity

**FIGURE 1:**
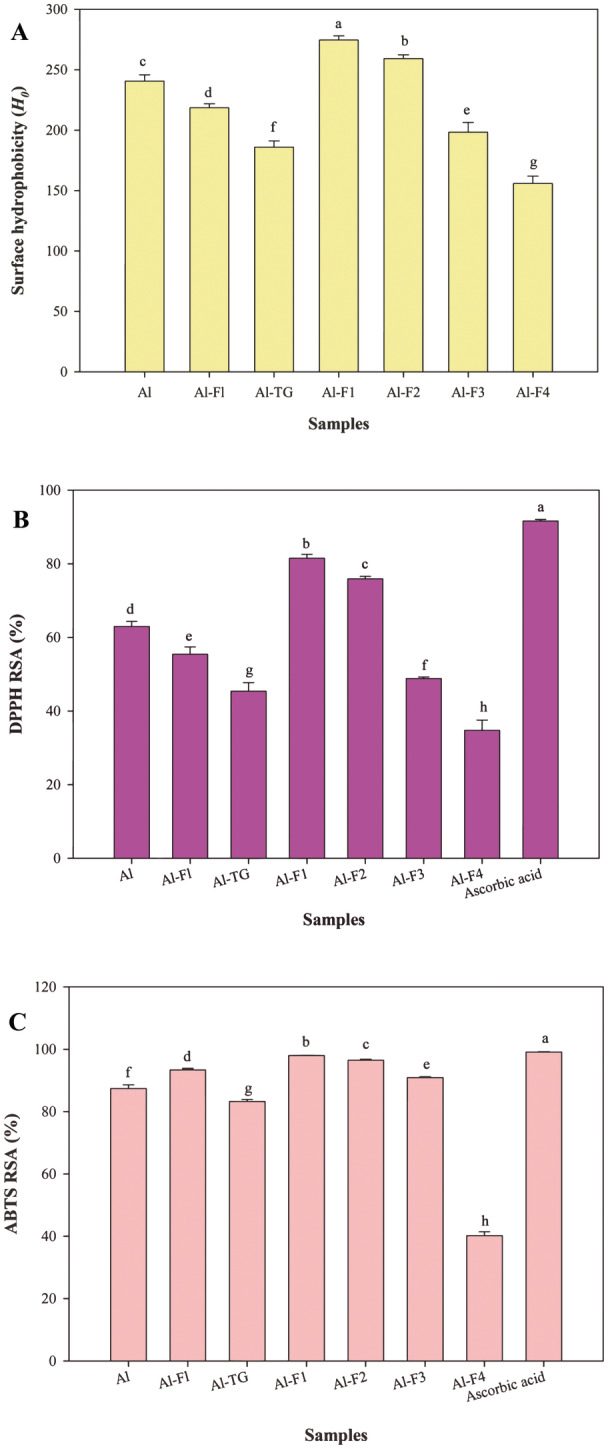
Surface hydrophobicity (*H*_0_) (A), DPPH RSA (%) (B), and ABTS^·+^ RSA (%) (C). Values of corn and soy mixture hydrolysate samples produced by Alcalase (Al), Alcalase‐Flavourzyme (Al‐FL), Alcalase‐Transglutaminase (Al‐TG), and ultrafiltration (Al‐F1, Al‐F2, Al‐F3, and Al‐F4). Data are means ± SD of three replicates.

The surface hydrophobicity (*H*_0_) is an important index for assessment of the biological and functional properties of proteins and peptides. The highest surface hydrophobicity was achieved for Al‐F1, while the lowest was observed for Al‐F4 (Figure 1A). The sequential hydrolysis and MTGase‐mediated cross‐linking of Al‐hydrolysate resulted in a decrease in *H*_0_ values. Subsequent hydrolysis of Al‐hydrolysates using Flavourzyme produced new peptides (Al‐FL) characterized by lower hydrophobic amino acid residues. On the other hand, cross‐linking process using MTGase resulted in the formation of peptides with buried hydrophobic patches. The fractions with MWs lower than 10 kDa had higher *H*_0_ values, which could be related to their high hydrophobic amino acid contents. Therefore, the surface hydrophobicity values correlated with the amino acid profiles. Similar results have been reported in previous studies (Liu, Fang, et al. 2020; Rahimi et al. 2022). Other studies have shown that corn proteins are inherently hydrophobic, thus the high hydrophobic amino acid contents (Jin et al. 2016). Enzymatic hydrolysis exposes the buried hydrophobic patches and then separation of peptides by ultrafiltration facilitates fractionation of the hydrophobic peptides (Bhadkaria et al. 2023; Zhu, He, and Hou 2019). Although SPH contains hydrophobic patches, the *H*_0_ value for CPH is significantly higher than that of SPH. Following the fractionation of SPH70:CPH30 (Al‐hydrolysates) by membrane ultrafiltration, Al‐F1 (UF fraction) exhibited a higher *H*_0_ value compared to other fractions and Al‐hydrolysates. This observation indicates that the process of fractionation by MW has the capacity to concentrate peptides with varying degrees of hydrophobicity and hydrophilicity.

### 3.3 SDS‐PAGE Analysis

**FIGURE 2:**
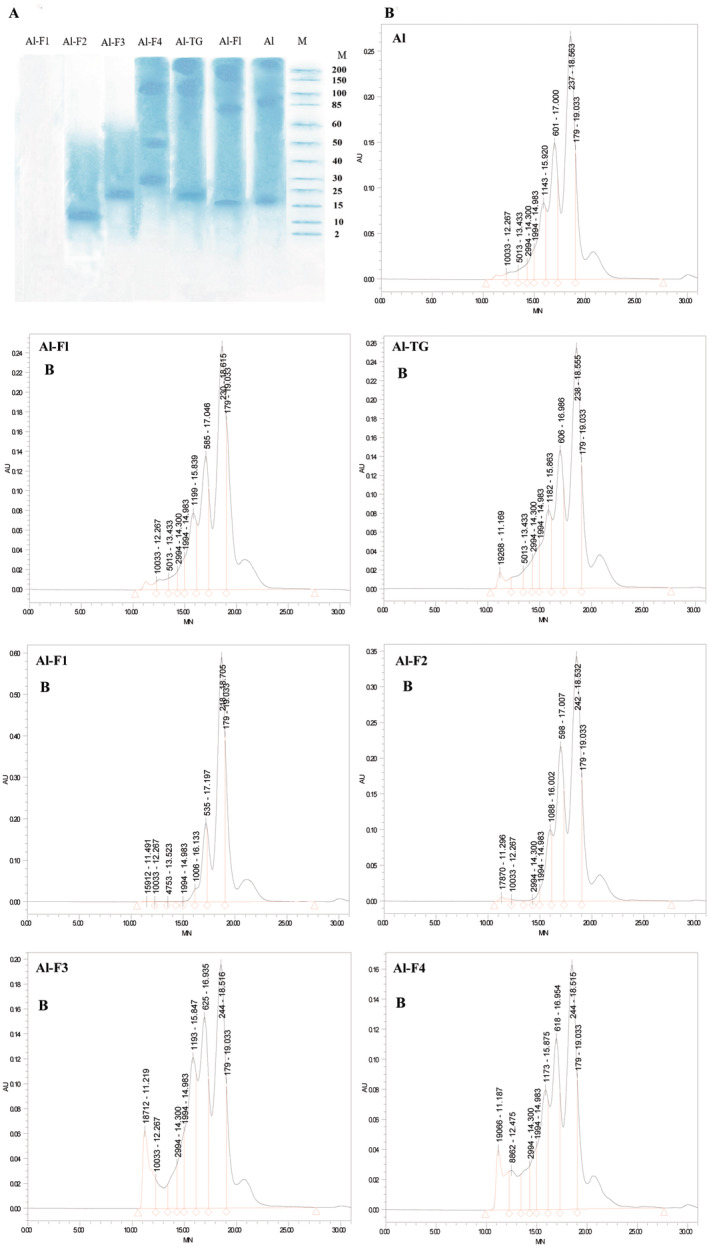
Electrophoresis MW profile (A) and Gel permeation chromatography (GPC) MW distribution (B) of corn and soy mixture hydrolysate samples produced by Alcalase (Al), Alcalase‐Flavourzyme (Al‐FL), Alcalase‐Transglutaminase (Al‐TG), and ultrafiltration (Al‐F1, Al‐F2, Al‐F3, and Al‐F4). Data are means ± SD of three replicates.

MW profiles of the modified Al‐hydrolysates are provided in Figure 2A. Three prominent bands were identified for Al‐hydrolysates (SPH70:CPH30), corresponding to peptides with MW of 200, 85–90, and 15–17 kDa. Sequential hydrolysis using Flavourzyme (Al‐FL) resulted in the development of smaller peptides with MW of 150, 70–80, and 15 kDa, accompanied by an increase in DH from 15% to 20%. On the other hand, MTGase‐mediated cross‐linking (Al‐TG) led to an increase in peptide MW. Notably, the bands initially observed at 15–17, 85–90, and 200 kDa shifted to 19, 100–120, and over 200 kDa, respectively. Indeed, cross‐linking enlarged the peptides through polymerization (Song et al. 2021). No band was detected for the fractionated peptides of Al‐F1 (UF fraction), likely due to the predominance of smaller peptides with MWs below 10 kDa; SDS‐PAGE is primarily suitable for the analysis of peptides with MWs exceeding 10 kDa (Fadimu et al. 2021). Al‐F2 had a prominent band at 10–12 kDa, Al‐F3 exhibited a distinct band corresponding to 20 kDa, and Al‐F4 displayed three prominent bands at 28–30, 50, and 100 kDa. Hence, the MW profiles for each fraction were consistent with the ultrafiltration membranes utilized for fractionation.

### 3.4 GPC Analysis

Gel permeation chromatography (GPC) is an effective technique for assessing the distribution of MWs, particularly for molecules with sizes below 10 kDa (Fadimu, Gill, et al. 2022). As depicted in Figure 2B and Table 2, the Al‐hydrolysates exhibited peptides with MWs ranging from 104 to 7094 Da. Sequential hydrolysis with Flavourzyme (Al‐FL) increased the proportion of small peptides, particularly the peak at 109 Da. This indicates that Flavourzyme produced smaller peptides and free amino acids, contributing to a meat‐like flavor and antioxidant activity (Wei, Thakur, Liu, Zhang, and Wei 2018). Al‐TG showed that contents of peptides with MW of 1385, 2429, 3834, and 7057 Da were increased after cross‐linking. Previous reports have indicated that cross‐linking process using MTGase promotes the presence of peptides with MWs of 2–5 kDa, which can increase the umami taste and improve the functional attributes (Rezvankhah et al. 2024). The peaks observed in Al‐F1 (UF fraction), particularly those associated with smaller peptides (111 and 263 Da), displayed higher areas. Notably, there was a significant decrease in areas related to the 1201 and 2362 Da peaks. On the other hand, in Al‐F2, the proportion of smaller/larger peptides decreased (105 and 283 Da), while the peaks corresponding to 682, 1297, 2305, 3785, and 7557 Da increased compared to those identified in Al‐F1 (UF fraction). Similar results were obtained for Al‐F3 and Al‐F4 while the areas of smaller peptides (102, 97, 291, and 287 Da) decreased remarkably. Additionally, the chromatogram analysis of Al‐F3 and Al‐F4 revealed that the content of larger peptides, particularly those with MWs of 2410, 2428, 3824, 3887, 7347, 7321, 16,140, and 16,084 Da, were significantly higher compared to other fractions, including Al‐hydrolysate, Al‐FL, and Al‐TG. This confirms that the ultrafiltration membranes facilitated the purification of peptides with distinct MWs, which determines the properties and specific applications of the peptides (He et al. 2013; Su et al. 2011).

**TABLE 2:** MW distribution of corn and soy mixture hydrolysate samples.

Sample^a^	MW (Da) (area (%))							
Al	104 (16.68)	281 (44.31)	690 (20.74)	1361 (11.22)	2402 (2.71)	3831 (1.86)	7094 (1.54)	14,748 (0.95)
Al‐FL	109 (23.10)	277 (39.65)	688 (18.65)	1369 (10.62)	2413 (2.78)	3838 (1.96)	7158 (1.88)	15,349 (1.36)
Al‐TG	99 (16.92)	281 (40.34)	691 (19.41)	1385 (11.29)	2429 (3.97)	3834 (3.19)	7057 (2.47)	16,117 (2.40)
Al‐F1	111 (28.64)	263 (57.19)	632 (12.63)	1201 (1.29)	2362 (0.03)	3981 (0.04)	7429 (0.08)	14,412 (0.1)
Al‐F2	105 (16.54)	283 (48.53)	682 (24.01)	1297 (9.11)	2305 (0.70)	3785 (0.19)	7557 (0.26)	15,443 (0.66)
Al‐F3	102 (11.49)	291 (32.40)	708 (21.39)	1383 (15.79)	2410 (4.74)	3824 (3.41)	7347 (3.11)	16,140 (7.68)
Al‐F4	97 (14.21)	287 (32.72)	702 (19.16)	1385 (13.16)	2428 (4.39)	3887 (4.13)	7321 (5.11)	16,084 (7.11)

^a^ Al‐hydrolysate was obtained through the mixing of soy protein hydrolysate (SPH:70) and corn protein hydrolysate (CPH:30). Al‐FL was sequential hydrolysis of SPH70:CPH30 using Flavourzyme. Al‐TG was sequential cross‐linking of SPH70:CPH30. Al‐F1, Al‐F2, Al‐F3, and Al‐F4 were fractionated hydrolysates obtained from Al‐hydrolysate, with MW of < 2, 2–10, 10–30, and 30–100 kDa, respectively. Data are means ± SD of three replicates.

### 3.5 In Vitro Biological Activity of Modified Hydrolysates

#### 3.5.1 DPPH RSA (%)

As shown in Figure 1B, Al‐F1 had the strongest DPPH RSA among the hydrolysates, followed by Al‐F2, Al, Al‐FL, Al‐F3, Al‐TG, and Al‐F4. The results showed a positive correlation between MWs achieved by SDS‐PAGE and GPC and antioxidant activity. Peptides with MW less than 10 kDa had the strongest antioxidant activity. This could be associated with either their high reactivity with DPPH radicals or their high surface hydrophobicity, which enabled favorable interaction with hydrophobic DPPH radicals (Moaveni et al. 2022). Further hydrolysis by Flavourzyme and cross‐linking by MTGase reduced the surface hydrophobicity of Al‐hydrolysates, leading to decreased DPPH radical scavenging activity (DPPH RSA). Sequential hydrolysis and MTGase‐mediated cross‐linking lowered surface hydrophobicity, which is closely related to DPPH RSA (Rezvankhah et al. 2023). Flavourzyme hydrolysis produced peptides with fewer hydrophobic amino acids. Additionally, further hydrolysis can separate antioxidant amino acids like tyrosine and phenylalanine or hydrophobic amino acids, resulting in reduced DPPH RSA (Suarez et al. 2021). The reduction in DPPH RSA of Al‐FL may also be due to reaggregation of peptides during Alcalase hydrolysis (Jin et al. 2016). MTGase‐mediated cross‐linking can rebury hydrophobic patches, decreasing surface hydrophobicity (Rezvankhah et al. 2024).

Fractionation of Al‐hydrolysates concentrated the smaller peptides with higher hydrophobic amino acid residues into fractions that efficiently interacted with DPPH radicals (Xie et al. 2024). Although the antioxidant activities of the hydrolysates were significantly lower (*p <* 0.05) than that of ascorbic acid (91.63%), fractionation improved the biological activities of Al‐hydrolysates. Al‐F1 with MW of < 2 kDa was comparable to other hydrolysates and approached the antioxidant capacity of ascorbic acid. Indeed, the main factor in increase of DPPH RSA of Al‐F1 (UF fraction) was increase in surface hydrophobicity that was resulted from the purification of peptides with high hydrophobic amino acids (Xu et al. 2021; Zhuang et al. 2024). Therefore, fractionation by membrane ultrafiltration can be considered an effective method for enhancing the antioxidant capacity of protein hydrolysates.

#### 3.5.2 ABTS^·+^ RSA (%)

ABTS^·+^ RSA of the hydrolysates are presented in Figure 1C. Among the hydrolysates, the highest antioxidant capacity was obtained for Al‐F1 (98.02%), followed by Al‐F2 (96.49%), Al‐FL (93.38%), Al‐F3 (90.95%), Al (87.40%), Al‐TG (83.26%), and Al‐F4 (40.18%). The results showed that, similar to the DPPH antioxidant results, peptides with lower MWs efficiently interacted with ABTS radicals, resulting in strong scavenging effects. Moreover, Al‐F1 had higher Tyr and Phe contents, which could contribute to stronger ABTS RSA. It was postulated that the fractionated peptides especially those derived from SPH had predominant effects on ABTS RSA which was in agreement with our recent study (Mirzaee et al. 2023). The SPH had more hydrophilic amino acids, thereby indicating stronger ABTS RSA than CPH. However, other fractions had higher hydrophilic amino acids, but the position of amino acids and MW of peptides could determine the final antioxidant activity (Tawalbeh, Ahmad, and Sarbon 2022).

#### 3.5.3 ACE‐Inhibitory Activity

**FIGURE 3:**
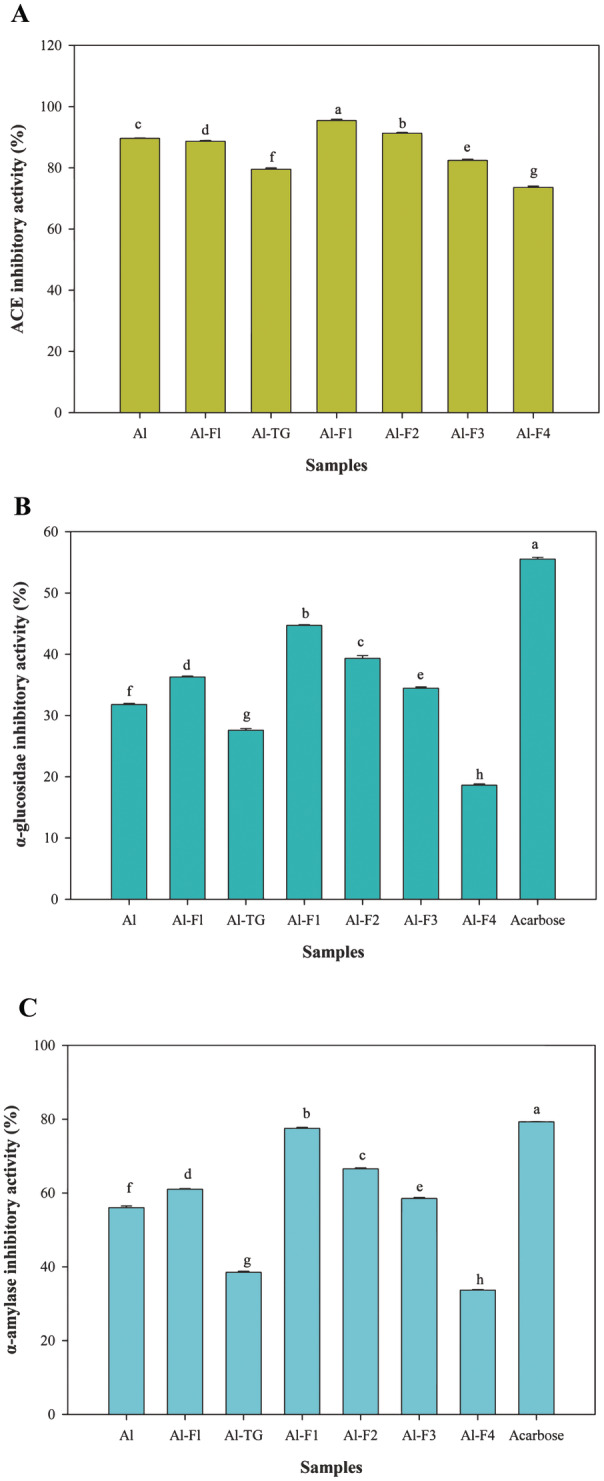
ACE (A), α‐glucosidase (B), and α‐amylase (C) inhibitory activities of corn and soy mixture hydrolysate samples produced by Alcalase (Al), Alcalase‐Flavourzyme (Al‐FL), Alcalase‐Transglutaminase (Al‐TG), and ultrafiltration (Al‐F1, Al‐F2, Al‐F3, and Al‐F4). Acarbose was used as positive control. Data are means ± SD of three replicates.

Fractionation of Al‐hydrolysates significantly increased ACE inhibition, which reached 95.45% and 91.31% for Al‐F1 and Al‐F2, respectively (*p* < 0.05) (Figure 3A). There are reports implying that peptides with a MW of < 1 kDa are the most active inhibitors, with inhibition of 81% ACE activity (Aondona et al. 2021). In addition, corn proteins hydrolyzed sequentially using Alcalase and Flavourzyme had 97.68% ACE inhibitory activity (Sharma et al. 2023). Our results indicate that sequential hydrolysis or cross‐linking reduced ACE‐inhibitory activities, which could be related to the reduction of hydrophobic patches that interact with ACE. Hydrophobic amino acid residues have significant effects on ACE‐inhibitory activity, due to predominant hydrophobic interactions of ACE active site (Wang et al. 2019). The hydrophobic amino acid residues on the enzyme active site are capable to interact with uncharged amino acids (Liu, Song, et al. 2020). Hence, when more hydrophobic peptides are produced, the hydrophobic–hydrophobic interactions are enhanced between the enzyme active site and peptides where Al‐F1 (UF fraction) was observed. Sequential hydrolysis by Flavourzyme and cross‐linking by MTGase reduced the hydrophobic patches, thus, reducing the ACE inhibition (Rezvankhah et al. 2024). Corn peptides have more hydrophobic amino acids than soy peptides. Sequential hydrolysis of SPH70:CPH30 might lead to separation of more hydrophobic peptides. Cross‐linking process using MTGase can rebury the unfolded peptide structure and reduce the surface hydrophobicity, thereby, reducing the hydrophobic interactions. On the other side, UF fractionation led to the purification of hydrophobic peptides (Al‐F1), and strong hydrophobic interactions were established. There is report implying that papain‐hydrolyzed soy protein isolate (SPI) and β‐conglycinin‐rich fraction developed by the papain hydrolysis exerted inhibition effects against the ACE two‐fold than SPI‐hydrolysate obtained by pepsin and/or glycinin‐rich fraction obtained by pepsin hydrolysis (Margatan et al. 2013). Also, β‐conglycinin was introduced as a potent precursor for ACE inhibition compared to glycinin (Margatan et al. 2013).

#### 3.5.4 α‐Glucosidase Inhibitory Activity

One of the important enzymes secreted by the pancreas is α‐glucosidase, which is associated with diabetes mellitus. The enzyme breaks down disaccharides into monosaccharides, predominantly glucose, which increases the glucose content of blood (Mirzaee et al. 2023). According to Figure 3B, the strongest α‐glucosidase inhibitory activity achieved for Al‐F1 (44.72%) (UF fraction), which was significantly higher than that of obtained for Al‐hydrolysates (SPH70:CPH30; 31.80%) (*p* < 0.05). Al‐F2 (39.34%), Al‐FL (36.30%) (sequential hydrolysate), Al‐F3 (34.47%), Al (31.80%), Al‐TG (27.60%), and Al‐F4 (18.62%) exhibited significantly lower α‐glucosidase inhibitory activity (*p* < 0.05). Acarbose, a commercial inhibitor, exhibited stronger α‐glucosidase inhibitory activity (55.54%) (*p* < 0.05). Fractionation of Al‐hydrolysates produced peptides with higher α‐glucosidase inhibitory activities. Particularly, Al‐F1 and Al‐F2 (UF fractions) with MWs of < 10 kDa had higher α‐glucosidase inhibitory activity, likely due to their high hydrophobic amino acid contents, which could efficiently interact with hydrophobic amino acid residues located at the active site of enzyme (Zheng et al. 2024). Recent reports have demonstrated that the peptides fraction from rice bran with MW of 1–3 kDa indicated the best α‐glucosidase inhibition activity. The findings of research study exhibited that rice bran peptides with MW of 1–3 kDa exhibited hypoglycemic effect on maltose tolerance test in mice. Alanine at C‐terminal and KK amino acid cluster played a key role in α‐glucosidase inhibition (Liu et al. 2024).

#### 3.5.5 α‐Amylase Inhibitory Activity

Another enzyme involved in glucose regulation is α‐amylase, which mainly breaks down starch to liberate smaller saccharides. Plant protein hydrolysates have shown promising α‐amylase inhibitory effects (Karimi, Azizi, and Ahmadi Gavlighi 2020; Karimi et al. 2020). As shown in Figure 3C, the sample with the highest α‐amylase inhibitory activity was Al‐F1 (77.52%) (UF fraction) followed by Al‐F2 (66.58%), Al‐FL (61.03%) (sequential hydrolysate), Al‐F3 (58.54%), Al (56.04%), and Al‐TG (38.51%). Acarbose inhibited α‐amylase activity by 79.30%, which was slightly higher than the effect of Al‐F1 fraction. Therefore, fractionation of Al‐hydrolysates effectively produced peptides with potent α‐amylase inhibitory activity. Peptides may bind enzyme allosteric sites, especially calcium and chloride ions (Karimi, Azizi, and Ahmadi Gavlighi 2020; Karimi et al. 2020). These interactions create unstable conformations, limiting enzyme–substrate binding. Additionally, residue hydrophobic amino acids may limit enzyme activity by interacting with its active site (Rodhi et al. 2024). Previous reports have mentioned that the peptide property of hydrophobic‐aliphatic is highly preferred in α‐amylase inhibition. Also, the position and type of amino acids in peptides have been highlighted. C…π interaction was mentioned to be essential in the hydrophobic interactions which are so vital in α‐amylase inhibition. Additionally, it was found that peptides with strong α‐amylase inhibition properties were highly hydrophobic and consisted of amino acids with aliphatic side chains, followed by neutral polar amino acids, irrespective of peptide length. Proline, leucine, and serine were identified as the top three reactive amino acid residues in these peptides, according to Rodhi et al. (2024).

### 3.6 Functional Properties of Modified Hydrolysates

#### 3.6.1 Solubility

The solubility of the modified hydrolysates is provided in Table 3. All samples had solubility higher than 90% at different pH values. This could be related to the unfolded structures of corn and soy peptides, exposed charged amino acids, and alterations of the hydrophobic/hydrophilic balance and MW. Several studies have reported that treatment of plant‐based protein hydrolysates by enzymatic and physical methods increases the solubility of hydrolysates (Liu et al. 2022). At pH 4.0 and 9.0, the highest solubility was obtained for Al‐FL (sequential hydrolysate), Al‐F1 (UF fraction), and Al‐F2. At pH 7.0, the highest solubility was obtained for Al‐FL (sequential hydrolysate), Al‐F1 (UF fraction), Al‐F2, and Al‐F3. Although the solubility of the modified hydrolysates was slightly higher at pH 7.0 and pH 9.0 compared to pH 4.0, due to exposed amino acids with negative charges on the surface of unfolded peptide chains, the alteration of solubility was negligible (Rezvankhah et al. 2021b). Moreover, there was a direct relationship between MW of peptides and solubility; as MW of peptides decreased, the solubility increased. Indeed, when the MW of peptides is decreased, more polar amino acids are located on the surface and polar–polar interactions with water are increased (Li et al. 2024). In recent reports, the solubility of wheat gluten was improved after hydrolysis by several proteases. Protease modification induced β‐sheet changes into α‐helix, β‐turn, and random coil. The proteases enhanced the combined effect of hydrogen bond and ionic bond in wheat gluten (Li et al. 2024).

**TABLE 3:** Functional properties of corn and soy mixture hydrolysate samples.

Sample^a^	Functional properties			
	EAI (m^2^/g)	ESI (min)	FC (%)	FS (%) after 30 min
Al	60.92 ± 0.42^b^	51.34 ± 0.23^b^	43.00 ± 0.31^b^	27.05 ± 0.48^b^
Al‐FL	55.42 ± 0.22^c^	45.38 ± 0.54^c^	40.34 ± 0.87^c^	25.05 ± 0.48^c^
Al‐TG	69.48 ± 0.16^a^	61.40 ± 0.65^a^	52.17 ± 1.20^a^	30.55 ± 0.91^a^
Al‐F1	40.54 ± 0.41^g^	36.45 ± 0.71^g^	26.53 ± 0.70^f^	14.72 ± 0.27^e^
Al‐F2	48.44 ± 0.68^e^	40.60 ± 0.84^e^	37.50 ± 1.21^d^	21.60 ± 0.60^d^
Al‐F3	50.74 ± 0.82^d^	42.43 ± 0.43^d^	38.67 ± 1.42^d^	21.77 ± 0.82^d^
Al‐F4	44.42 ± 0.62^f^	39.14 ± 0.22^f^	29.54 ± 0.31^e^	15.14 ± 0.84^e^

Sample	Solubility (%)		
	pH = 4	pH = 7	pH = 9
Al	94.00 ± 0.30^c^	96.50 ± 0.20^b^	98.00 ± 0.50^b^
Al‐FL	98.00 ± 0.50^a^	100.0 ± 0.00^a^	100.0 ± 0.00^a^
Al‐TG	92.00 ± 0.50^d^	95.00 ± 0.50^c^	97.00 ± 0.40^c^
Al‐F1	98.00 ± 0.50^a^	100.0 ± 0.00^a^	100.0 ± 0.00^a^
Al‐F2	98.00 ± 0.50^a^	100.0 ± 0.00^a^	100.0 ± 0.00^a^
Al‐F3	97.00 ± 0.30^b^	100.0 ± 0.00^a^	98.00 ± 0.50^b^
Al‐F4	94.00 ± 0.60^c^	96.60 ± 0.50^b^	98.00 ± 0.50^b^

*Note:* Different small letters in each column indicate significant differences (*p* < 0.05).

^a^ Al‐hydrolysate was obtained through the mixing of soy protein hydrolysate (SPH:70) and corn protein hydrolysate (CPH:30). Al‐FL was sequential hydrolysis of SPH70:CPH30 using Flavourzyme. Al‐TG was sequential cross‐linking of SPH70:CPH30. Al‐F1, Al‐F2, Al‐F3, and Al‐F4 were fractionated hydrolysates obtained from Al‐hydrolysate, with MW of < 2, 2–10, 10–30, and 30–100 kDa, respectively. Data are means ± SD of three replicates.

#### 3.6.2 Emulsion Activity Index and Emulsion Stability Index

EAI and ESI of modified hydrolysates are presented in Table 3. The highest EAI value (69.48 m^2^/g) was obtained for Al‐TG, while the lowest value (40.54 m^2^/g) was achieved for Al‐F1. Similar trends were observed for ESI values. EAI and ESI depend on factors like amino acid composition, MW, hydrophilic/hydrophobic balance, and conformation, with the hydrophilic/hydrophobic balance being the most crucial (Rezvankhah, Yarmand, and Ghanbarzadeh 2022). Corn peptides are more hydrophobic, while soy peptides are more hydrophilic (Mirzaee et al. 2023). Combining these peptides produces mixed hydrolysates with improved EAI and ESI due to balanced residues. Similarly, cross‐linking with MTGase increases EAI and ESI values. MTGase facilitates transamination reactions to catalyze inter‐ and intramolecular cross‐linking of peptides (Tian et al. 2024; Vijayan et al. 2024), increasing negative surface charge and enhancing functional properties (Vijayan et al. 2024). MTGase‐induced cross‐linking restores the emulsifying and foaming properties of SPHs, but high concentrations (5 U/g protein) can reduce EAI due to aggregation of high‐MW peptides. MTGase rearranges hydrophobic/hydrophilic residues in tertiary structures, yielding peptides with enhanced amphiphilic nature at interfaces (Zhang et al. 2021). Conversely, sequential hydrolysis by Flavourzyme and ultrafiltration decreased EAI and ESI; the Al‐F1 fraction showed the lowest values, suggesting that lower‐MW peptides have weaker functional properties, likely due to altered hydrophobic/hydrophilic balance.

#### 3.6.3 Foaming Capacity and Stability

FC and FS values of hydrolysate and peptide samples are provided in Table 3. The sequential hydrolysis and fractionation decreased the FC and FS, while MTGase‐mediated cross‐linking significantly increased the respective values (*p* < 0.05). The hydrophobic/hydrophilic balance is crucial for FC and FS values. Low‐MW peptides rapidly adsorb at the air–water interface, but their insufficient coating and aggregation lead to low FC and FS (Rezvankhah et al. 2024). Peptides produced by MTGase show significantly higher FC and FS (*p* < 0.05) due to better coating of air molecules and more resistant film formation. MTGase‐induced cross‐linking rearranges hydrophobic/hydrophilic residues, enhancing the peptides' amphiphilic nature and stability at the interface. Improved hydrophobic/hydrophilic balance enhances FC and FS, as cross‐linked peptides act as emulsifiers interacting with water and oxygen molecules. Sequential hydrolysis with Flavourzyme (Al‐FL) alters the hydrophobic/hydrophilic balance, affecting the interfacial properties of peptides. Similarly, fractions from the UF process may have imbalanced hydrophobic/hydrophilic amino acids, impacting their interfacial properties (Zhang et al. 2021).

### 3.7 FTIR Analysis

**FIGURE 4:**
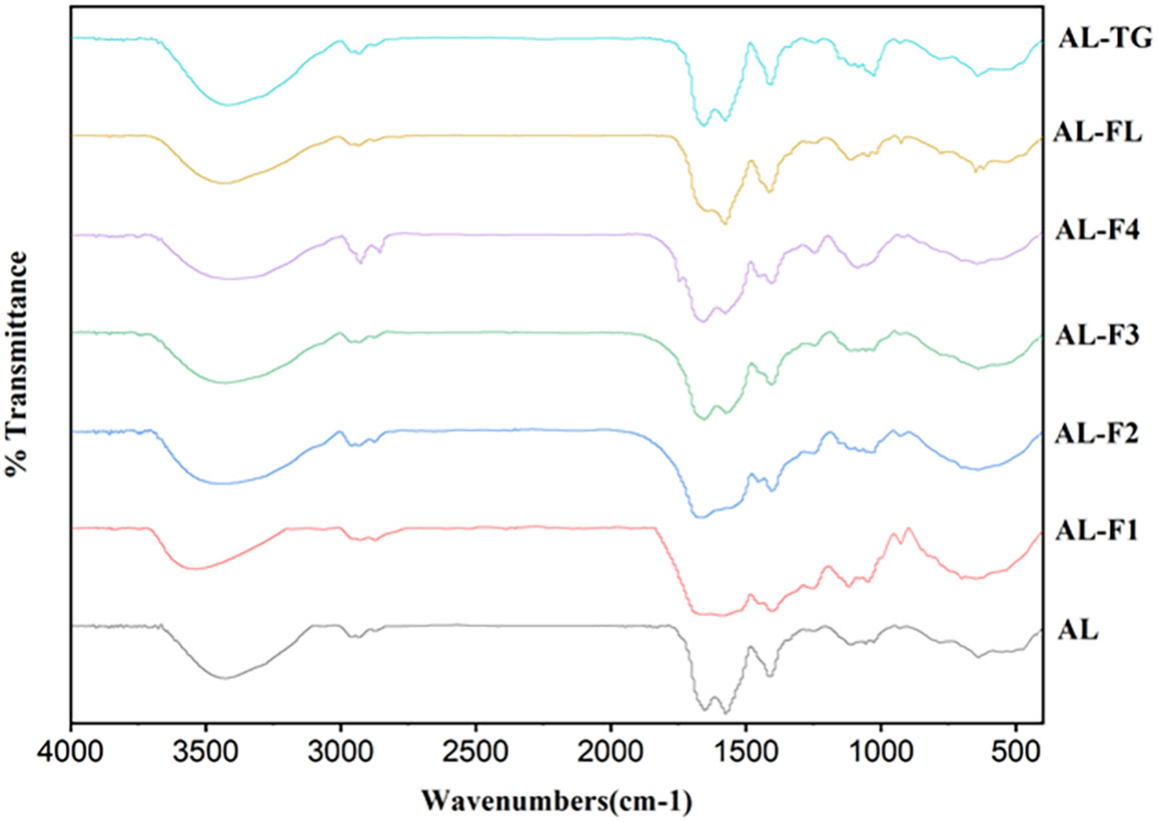
FTIR spectrums of corn and soy mixed hydrolysates produced by Alcalase (Al), Alcalase‐Flavourzyme (Al‐FL), Alcalase‐Transglutaminae (Al‐TG), and ultrafiltration (Al‐F1, Al‐F2, Al‐F3, and Al‐F4).

FTIR spectra of the hydrolysates and peptides are shown in Figure 4. A broad stretching peak was appeared for Al‐hydrolysates at 3423 cm^−1^. Another vibration peak at stretching status was detected at 1651 cm^−1^ and a bending peak was observed at 1573 cm^−1^; both peaks enabled the analysis of the amide bands (I and II) alterations. Amide I arises from the stretching of the carbonyl (C=O) group, whereas amide II is attributed to the bending of N–H and stretching of CH vibrations (Mattice and Marangoni 2021). The spectrum of Al‐FL (sequential hydrolysate) indicated low‐intensity peak at 1651 cm^−1^, which implies that amide I was efficiently influenced after sequential hydrolysis. Cross‐linking process using MTGase resulted to an increase in frequency of peak detected at 3421 cm^−1^, attributing to noncovalent hydrogen bond formation (Rezvankhah et al. 2021a). This could be the main reason for the highest EAI, ESI, FC, and FS values observed for Al‐TG. Also, the vibration that appeared at 1652 cm^−1^ had slightly higher frequency while partially shifting from 1573 to 1575 cm^−1^. The values of identifying bands used include intermolecular β‐sheets at 1630–1640 cm^−1^, random coil at 1640–1648 cm^−1^, and α‐helices at 1648–1658 cm^−1^. According to Mattice and Marangoni (2021), cross‐linking of zein using MTGase resulted in an increase in intermolecular β‐sheets and random coils, and a reduction in α‐helical structures. The proportional proportions of each secondary structure did not alter, indicating that MTGase had modest impact on the secondary structure, as validated by deconvolution. The UF peptide fraction of Al‐F1 indicated that most probably the secondary structure disappeared, amide bands were detected with low intensity. Al‐F2 showed a peak at 1669 cm^−1^ which could be due to β‐turns (Mattice and Marangoni 2021). Al‐F3 and Al‐F4 exhibited peaks at 1653 and 1570 cm^−1^ and 1654 and 1575 cm^−1^, suggesting that the peptides with high MWs maintained their secondary structures.

Regarding the secondary structure in amid I region (Table 4), second hydrolysis of Al‐hydrolysates led to reduction of α‐helix, increase in β‐Sheet, and reduction of β‐turn. Cross‐linking process using MTGase resulted in an increase in α‐helix, reduction of β‐Sheet, and increase in β‐turn. Thus, cross‐linking process had inverse effects on Al‐hydrolysates compared to sequential hydrolysis by Flavourzyme. In terms of fractions, Al‐F1 exhibited reduction of α‐helix, increase in β‐Sheet, and slight increase in β‐turn. Al‐F2 exhibited slight increase in α‐helix, slight increase in β‐Sheet, and increase in β‐turn. Al‐F3 exhibited reduction of α‐helix, slight reduction of β‐Sheet, and increase in β‐turn. Al‐F4 exhibited increase in α‐helix, slight reduction of β‐Sheet, and increase in β‐turn. Hence, further processing the Al‐hydrolysates changed the secondary structure in amide region (Zhang et al. 2022).

**TABLE 4:** Secondary structure in amide I region (1600–1700 cm^−1^) of corn and soy mixture hydrolysate samples by FTIR analysis.

Sample	Area (%)						
	α‐helix	β‐Sheet			β‐Turns		
	1650–1660 cm^−1^	1610–1640 cm^−1^	1670–1690 cm^−1^	Total	1660–1670 cm^−1^	1690–1700 cm^−1^	Total
AL	14.97	31.35	16.26	47.62	12.16	2.19	14.35
Al‐F1	10.75	33.55	18.80	52.36	10.50	4.43	14.94
Al‐F2	15.23	19.71	29.18	48.89	15.70	6.30	22.01
Al‐F3	16.41	24.32	22.35	46.64	14.85	3.80	18.66
Al‐F4	17.23	21.79	24.31	46.10	15.93	4.19	20.12
Al‐FL	11.18	37.38	14.69	52.08	9.92	2.59	12.52
Al‐TG	16.02	26.85	19.22	46.08	14.25	2.64	16.89

*Note:* Al‐hydrolysate was obtained through the mixing of soy protein hydrolysate (SPH:70) and corn protein hydrolysate (CPH:30). Al‐FL was sequential hydrolysis of SPH70:CPH30 using Flavourzyme. Al‐TG was sequential cross‐linking of SPH70:CPH30. Al‐F1, Al‐F2, Al‐F3, and Al‐F4 were fractionated hydrolysates obtained from Al‐hydrolysate, with MW of < 2, 2–10, 10–30, and 30–100 kDa, respectively. Data are means ± SD of three replicates.

## 4 Conclusions

New formulated mixed corn and soy peptides were modified by Alcalase, Flavourzyme, MTGase, and ultrafiltration. Ultrafiltration fractions including Al‐F1 and Al‐F2 indicated the highest *H*_0_, in vitro antioxidant, antihypertensive, and antidiabetic activities, which were attributed to their balanced hydrophobic and hydrophilic amino acid contents. Al‐TG showed the highest EAI, ESI, FC, and FS, implying that cross‐linking can be considered an effective approach for improving the functional properties of peptides. Therefore, fractionation by membrane ultrafiltration can be introduced as an effective technique to produce potent biologically active peptides while MTGase‐mediated cross‐linking can produce peptides with improved functional attributes. In the present study, Al‐F1 as UF fraction and Al‐TG were introduced as the strongest mixed peptides from biological and functional side of views.

## Author Contributions

**Homaira Mirzaee:** data curation (equal), investigation (equal), methodology (equal), writing – original draft (equal). **Hassan Ahmadi Gavlighi:** supervision (lead), writing – review and editing (equal). **Mehdi Nikoo:** investigation (equal), methodology (equal), writing – review and editing (equal). **Chibuike C. Udenigwe:** supervision (equal), writing – review and editing (equal). **Amir Rezvankhah:** data curation (equal), investigation (equal), methodology (equal), writing – original draft (equal). **Faramarz Khodaiyan:** writing – review and editing (equal).

## Ethics Statement

This study does not involve any human or animal testing.

## Conflicts of Interest

The authors declare no conflicts of interest.

## Data Availability

Data will be made available on request.

## References

[fsn34532-bib-0001] Aondona, M. M. , J. K. Ikya , M. T. Ukeyima , T. W. J. A. Gborigo , R. E. Aluko , and A. T. Girgih . 2021. “In Vitro Antioxidant and Antihypertensive Properties of Sesame Seed Enzymatic Protein Hydrolysate and Ultrafiltration Peptide Fractions.” Journal of Food Biochemistry 45, no. 1: e13587. 10.1111/jfbc.13587. 33346921

[fsn34532-bib-0002] Bhadkaria, A. , D. T. Narvekar , D. P. Nagar , S. P. Sah , N. Srivastava , and S. S. Bhagyawant . 2023. “Purification, Molecular Docking and *In Vivo* Analyses of Novel Angiotensin‐Converting Enzyme Inhibitory Peptides From Protein Hydrolysate of Moth Bean (*Vigna aconitifolia* (Jacq.) Màrechal) Seeds.” International Journal of Biological Macromolecules 230: 123138. 10.1016/J.IJBIOMAC.2023.123138. 36610577

[fsn34532-bib-0003] Bradford, M. M. 1976. “A Rapid and Sensitive Method for the Quantitation of Microgram Quantities of Protein Utilizing the Principle of Protein‐Dye Binding.” Analytical Biochemistry 72, no. 1–2: 248–254. 942051 10.1016/0003-2697(76)90527-3

[fsn34532-bib-0004] Dabbour, M. , R. He , B. Mintah , M. K. Golly , and H. Ma . 2020. “Ultrasound Pretreatment of Sunflower Protein: Impact on Enzymolysis, ACE‐Inhibition Activity, and Structure Characterization.” Journal of Food Processing and Preservation 44, no. 4: e14398. 10.1111/jfpp.14398.

[fsn34532-bib-0005] Daliri, H. , R. Ahmadi , A. Pezeshki , et al. 2021. “Quinoa Bioactive Protein Hydrolysate Produced by Pancreatin Enzyme‐Functional and Antioxidant Properties.” LWT ‐ Food Science and Technology 150: 111853. 10.1016/J.LWT.2021.111853.

[fsn34532-bib-0006] de Matos, F. M. , J. T. J. G. de Lacerda , G. Zanetti , and R. J. S. de Castro . 2022. “Production of Black Cricket Protein Hydrolysates With α‐Amylase, α‐Glucosidase and Angiotensin I‐Converting Enzyme Inhibitory Activities Using a Mixture of Proteases.” Biocatalysis and Agricultural Biotechnology 39: 102276. 10.1016/J.BCAB.2022.102276.

[fsn34532-bib-0007] Fadimu, G. J. , A. Farahnaky , H. Gill , and T. Truong . 2022. “Influence of Ultrasonic Pretreatment on Structural Properties and Biological Activities of Lupin Protein Hydrolysate.” International Journal of Food Science & Technology 57, no. 3: 1729–1738. 10.1111/IJFS.15549.

[fsn34532-bib-0008] Fadimu, G. J. , H. Gill , A. Farahnaky , and T. Truong . 2021. “Investigating the Impact of Ultrasound Pretreatment on the Physicochemical, Structural, and Antioxidant Properties of Lupin Protein Hydrolysates.” Food and Bioprocess Technology 14, no. 11: 2004–2019. 10.1007/s11947-021-02700-4.

[fsn34532-bib-0009] Fadimu, G. J. , H. Gill , A. Farahnaky , and T. Truong . 2022. “Improving the Enzymolysis Efficiency of Lupin Protein by Ultrasound Pretreatment: Effect on Antihypertensive, Antidiabetic and Antioxidant Activities of the Hydrolysates.” Food Chemistry 383: 132457. 10.1016/j.foodchem.2022.132457. 35183959

[fsn34532-bib-0010] He, R. , A. Alashi , S. A. Malomo , et al. 2013. “Antihypertensive and Free Radical Scavenging Properties of Enzymatic Rapeseed Protein Hydrolysates.” Food Chemistry 141, no. 1: 153–159. 10.1016/j.foodchem.2013.02.087. 23768341

[fsn34532-bib-0011] Hu, R. , J. Xu , G. Qi , W. Wang , X. S. Sun , and Y. Li . 2022. “Antioxidative Hydrolysates From Corn Gluten Meal May Effectively Reduce Lipid Oxidation and Inhibit HepG2 Cancer Cell Growth.” Journal of Agriculture and Food Research 7: 100252. 10.1016/j.jafr.2021.100252.

[fsn34532-bib-0012] Jin, J. , H. Ma , B. Wang , et al. 2016. “Effects and Mechanism of Dual‐Frequency Power Ultrasound on the Molecular Weight Distribution of Corn Gluten Meal Hydrolysates.” Ultrasonics Sonochemistry 30: 44–51. 10.1016/J.ULTSONCH.2015.11.021. 26703201

[fsn34532-bib-0013] Jin, J. , H. Ma , C. Zhou , et al. 2015. “Effect of Degree of Hydrolysis on the Bioavailability of Corn Gluten Meal Hydrolysates.” Journal of the Science of Food and Agriculture 95, no. 12: 2501–2509. 10.1002/JSFA.6982. 25367020

[fsn34532-bib-0014] Karimi, A. , M. H. Azizi , and H. Ahmadi Gavlighi . 2020. “Fractionation of Hydrolysate From Corn Germ Protein by Ultrafiltration: *In Vitro* Antidiabetic and Antioxidant Activity.” Food Science & Nutrition 8, no. 5: 2395–2405. 10.1002/fsn3.1529. 32405396 PMC7215226

[fsn34532-bib-0015] Karimi, N. , M. Nikoo , H. A. Gavlighi , S. P. Gheshlaghi , J. M. Regenstein , and X. Xu . 2020. “Effect of Pacific White Shrimp (*Litopenaeus vannamei*) Protein Hydrolysates (SPH) and (−)‐epigallocatechin Gallate (EGCG) on Sourdough and Bread Quality.” LWT ‐ Food Science and Technology 131: 109800.

[fsn34532-bib-0016] Li, W. , Q. Zhou , J. Xu , et al. 2024. “Insight Into the Solubilization Mechanism of Wheat Gluten by Protease Modification From Conformational Change and Molecular Interaction Perspective.” Food Chemistry 447: 138992. 10.1016/J.FOODCHEM.2024.138992. 38503066

[fsn34532-bib-0017] Liu, P. , M. Huang , S. Song , et al. 2012. “Sensory Characteristics and Antioxidant Activities of Maillard Reaction Products From Soy Protein Hydrolysates With Different Molecular Weight Distribution.” Food and Bioprocess Technology 5, no. 5: 1775–1789. 10.1007/s11947-010-0440-3.

[fsn34532-bib-0018] Liu, W. , R. Ma , G. Cui , et al. 2024. “Rice Bran Peptide With α‐Glucosidase Inhibition Activity: Preparation, Evaluation and Molecular Mechanism.” Journal of Cereal Science 115: 103837. 10.1016/J.JCS.2023.103837.

[fsn34532-bib-0019] Liu, W. Y. , L. Fang , X. W. Feng , G. M. Li , and R. Z. Gu . 2020. “ *In Vitro* Antioxidant and Angiotensin I‐Converting Enzyme Inhibitory Properties of Peptides Derived From Corn Gluten Meal.” European Food Research and Technology 246, no. 10: 2017–2027. 10.1007/S00217-020-03552-6.

[fsn34532-bib-0020] Liu, X. , J. Wang , Y. Liu , N. Cui , D. Wang , and X. Zheng . 2022. “Conjugation of the Glutelin Hydrolysates‐Glucosamine by Transglutaminase and Functional Properties and Antioxidant Activity of the Products.” Food Chemistry 380: 132210. 10.1016/J.FOODCHEM.2022.132210. 35093648

[fsn34532-bib-0021] Liu, X. L. , C. L. Song , J. P. Chen , X. Liu , J. Ren , and X. Q. Zheng . 2020. “Preparation and Evaluation of New Glycopeptides Obtained by Proteolysis From Corn Gluten Meal Followed by Transglutaminase‐Induced Glycosylation With Glucosamine.” Food 9, no. 5: 555. 10.3390/foods9050555. PMC7278801 32370047

[fsn34532-bib-0022] Margatan, W. , K. Ruud , Q. Wang , T. Markowski , and B. Ismail . 2013. “Angiotensin Converting Enzyme Inhibitory Activity of Soy Protein Subjected to Selective Hydrolysis and Thermal Processing.” Journal of Agricultural and Food Chemistry 61, no. 14: 3460–3467. 10.1021/JF4001555/ASSET/IMAGES/MEDIUM/JF-2013-001555_0004.GIF. 23514371

[fsn34532-bib-0023] Mattice, K. D. , and A. G. Marangoni . 2021. “Physical Properties of Zein Networks Treated With Microbial Transglutaminase.” Food Chemistry 338: 128010. 10.1016/J.FOODCHEM.2020.128010. 32932084

[fsn34532-bib-0024] Mirzaee, H. , H. Ahmadi Gavlighi , M. Nikoo , C. C. Udenigwe , and F. Khodaiyan . 2023. “Relation of Amino Acid Composition, Hydrophobicity, and Molecular Weight With Antidiabetic, Antihypertensive, and Antioxidant Properties of Mixtures of Corn Gluten and Soy Protein Hydrolysates.” Food Science & Nutrition 11, no. 3: 1257–1271. 10.1002/fsn3.3160. 36911847 PMC10003021

[fsn34532-bib-0025] Moaveni, S. , M. Salami , M. Khodadadi , M. McDougall , and Z. Emam‐Djomeh . 2022. “Investigation of *S. limacinum* Microalgae Digestibility and Production of Antioxidant Bioactive Peptides.” LWT ‐ Food Science and Technology 154: 112468. 10.1016/J.LWT.2021.112468.

[fsn34532-bib-0026] Nikoo, M. , S. Benjakul , M. Yasemi , H. A. Gavlighi , and X. Xu . 2019. “Hydrolysates From Rainbow Trout (*Oncorhynchus mykiss*) Processing By‐Product With Different Pretreatments: Antioxidant Activity and Their Effect on Lipid and Protein Oxidation of Raw Fish Emulsion.” LWT ‐ Food Science and Technology 108: 120–128.

[fsn34532-bib-0027] Phongthai, S. , S. D'Amico , R. Schoenlechner , W. Homthawornchoo , and S. Rawdkuen . 2018. “Fractionation and Antioxidant Properties of Rice Bran Protein Hydrolysates Stimulated by In Vitro Gastrointestinal Digestion.” Food Chemistry 240: 156–164. 10.1016/j.foodchem.2017.07.080. 28946256

[fsn34532-bib-0028] Rahimi, R. , H. Ahmadi Gavlighi , R. Amini Sarteshnizi , M. Barzegar , and C. C. Udenigwe . 2022. “In Vitro Antioxidant Activity and Antidiabetic Effect of Fractionated Potato Protein Hydrolysate Via Ultrafiltration and Adsorption Chromatography.” LWT ‐ Food Science and Technology 154: 112765. 10.1016/j.lwt.2021.112765.

[fsn34532-bib-0029] Rezvankhah, A. , B. Ghanbarzadeh , H. Mirzaee , A. Ahmadi Hassan Abad , A. Tavakkoli , and A. Yarmand . 2024. “Conjugation of Gum Arabic and Lentil Protein Hydrolysates Through Maillard Reaction: Antioxidant Activity, Volatile Compounds, Functional and Sensory Properties.” Food Science & Nutrition 12: 1–19. 10.1002/fsn3.3966. PMC11016417 38628169

[fsn34532-bib-0030] Rezvankhah, A. , M. S. Yarmand , and B. Ghanbarzadeh . 2022. “The Effects of Combined Enzymatic and Physical Modifications of Lentil Protein Applying Alcalase, Flavourzyme, Microbial Transglutaminase, and Ultrasound: Antioxidant, Antihypertension, and Antidiabetic Activities.” Journal of Food Measurement and Characterization 16, no. 5: 3743–3759. 10.1007/s11694-022-01478-z.

[fsn34532-bib-0031] Rezvankhah, A. , M. S. Yarmand , B. Ghanbarzadeh , and H. Mirzaee . 2021a. “Characterization of Bioactive Peptides Produced From Green Lentil (*Lens culinaris*) Seed Protein Concentrate Using Alcalase and Flavourzyme in Single and Sequential Hydrolysis.” Journal of Food Processing and Preservation 45, no. 11: e15932. 10.1111/jfpp.15932.

[fsn34532-bib-0032] Rezvankhah, A. , M. S. Yarmand , B. Ghanbarzadeh , and H. Mirzaee . 2021b. “Generation of Bioactive Peptides From Lentil Protein: Degree of Hydrolysis, Antioxidant Activity, Phenol Content, ACE‐Inhibitory Activity, Molecular Weight, Sensory, and Functional Properties.” Journal of Food Measurement and Characterization 15, no. 6: 5021–5035. 10.1007/s11694-021-01077-4.

[fsn34532-bib-0033] Rezvankhah, A. , M. S. Yarmand , B. Ghanbarzadeh , and H. Mirzaee . 2023. “Development of Lentil Peptides With Potent Antioxidant, Antihypertensive, and Antidiabetic Activities Along With Umami Taste.” Food Science & Nutrition 11, no. 6: 2974–2989. 10.1002/fsn3.3279. 37324857 PMC10261806

[fsn34532-bib-0034] Rodhi, A. M. , P. G. Yap , O. A. Olalere , and C. Y. Gan . 2024. “Unveiling α‐Amylase Inhibition: A Bioinformatics Perspective on Peptide Properties and Amino Acid Contributions.” Journal of Molecular Structure 1305: 137768. 10.1016/J.MOLSTRUC.2024.137768.

[fsn34532-bib-0035] Sharma, S. , R. Pradhan , A. Manickavasagan , A. Tsopmo , M. Thimmanagari , and A. Dutta . 2023. “Corn Distillers Solubles by Two‐Step Proteolytic Hydrolysis as a New Source of Plant‐Based Protein Hydrolysates With ACE and DPP4 Inhibition Activities.” Food Chemistry 401: 134120. 10.1016/J.FOODCHEM.2022.134120. 36096002

[fsn34532-bib-0036] Shi, A. M. , B. Jiao , H. Z. Liu , et al. 2018. “Effects of Proteolysis and Transglutaminase Crosslinking on Physicochemical Characteristics of Walnut Protein Isolate.” LWT ‐ Food Science and Technology 97: 662–667. 10.1016/j.lwt.2018.07.043.

[fsn34532-bib-0037] Singh, U. , D. Kaur , V. Mishra , and M. Krishania . 2022. “Combinatorial Approach to Prepare Antioxidative Protein Hydrolysate From Corn Gluten Meal With Dairy Whey: Preparation, Kinetics, Nutritional Study and Cost Analysis.” LWT ‐ Food Science and Technology 153: 112437.

[fsn34532-bib-0038] Song, C. , X. Sun , J. Yang , et al. 2021. “TGase‐Induced Glycosylated Soy Protein Products With Limited Enzymatic Hydrolysis Showed Enhanced Foaming Property.” European Food Research and Technology 247, no. 10: 2557–2563. 10.1007/S00217-021-03815-W.

[fsn34532-bib-0039] Song, N. , C. Tan , M. Huang , et al. 2013. “Transglutaminase Cross‐Linking Effect on Sensory Characteristics and Antioxidant Activities of Maillard Reaction Products From Soybean Protein Hydrolysates.” Food Chemistry 136, no. 1: 144–151. 10.1016/j.foodchem.2012.07.100. 23017405

[fsn34532-bib-0040] Su, G. , L. Zheng , C. Cui , B. Yang , J. Ren , and M. Zhao . 2011. “Characterization of Antioxidant Activity and Volatile Compounds of Maillard Reaction Products Derived From Different Peptide Fractions of Peanut Hydrolysate.” Food Research International 44, no. 10: 3250–3258. 10.1016/J.FOODRES.2011.09.009.

[fsn34532-bib-0041] Suarez, L. M. , H. Fan , J. E. Zapata , and J. Wu . 2021. “Optimization of Enzymatic Hydrolysis for Preparing Cassava Leaf Hydrolysate With Antioxidant Activity.” Food and Bioprocess Technology 14, no. 12: 2181–2194. 10.1007/S11947-021-02693-0/METRICS.

[fsn34532-bib-0042] Tawalbeh, D. , W. A. N. W. Ahmad , and N. M. Sarbon . 2022. “Effect of ultrasound pretreatment on the functional and bioactive properties of legumes protein hydrolysates and peptides: A comprehensive review.” Critical Reviews in Food Science and Nutrition 64: 2548–2578. 10.1080/87559129.2022.2069258.36200775

[fsn34532-bib-0043] Tian, Y. , S. Wang , J. Lv , M. Ma , Y. Jin , and X. Fu . 2024. “Transglutaminase Cross‐Linking Ovalbumin‐Flaxseed Oil Emulsion Gels: Properties, Microstructure, and Performance in Oxidative Stability.” Food Chemistry 448: 138988. 10.1016/J.FOODCHEM.2024.138988. 38522295

[fsn34532-bib-0044] Vijayan, P. , Z. Song , J. Y. H. Toy , L. L. Yu , and D. Huang . 2024. “Effect of Transglutaminase on Gelation and Functional Proteins of Mung Bean Protein Isolate.” Food Chemistry 454: 139590. 10.1016/J.FOODCHEM.2024.139590. 38823202

[fsn34532-bib-0045] Wang, K. , S. Luo , J. Cai , et al. 2016. “Effects of Partial Hydrolysis and Subsequent Cross‐Linking on Wheat Gluten Physicochemical Properties and Structure.” Food Chemistry 197: 168–174. 10.1016/J.FOODCHEM.2015.10.123. 26616937

[fsn34532-bib-0046] Wang, R. , H. Zhao , X. Pan , C. Orfila , W. Lu , and Y. Ma . 2019. “Preparation of Bioactive Peptides With Antidiabetic, Antihypertensive, and Antioxidant Activities and Identification of α‐Glucosidase Inhibitory Peptides From Soy Protein.” Food Science & Nutrition 7, no. 5: 1848–1856. 10.1002/FSN3.1038. 31139399 PMC6526634

[fsn34532-bib-0058] Wei, C.‐K. , K. Thakur , D.‐H. Liu , J.‐G. Zhang , and Z.‐J. Wei . 2018. “Enzymatic Hydrolysis of Flaxseed (*Linum usitatissimum* L.) Protein and Sensory Characterization of Maillard Reaction Products.” Food Chemistry 263: 186–193. 10.1016/j.foodchem.2018.04.120. 29784306

[fsn34532-bib-0047] Xie, M. , Y. Ma , F. An , et al. 2024. “Ultrasound‐Assisted Fermentation for Antioxidant Peptides Preparation From Okara: Optimization, Stability, and Functional Analyses.” Food Chemistry 439: 138078. 10.1016/J.FOODCHEM.2023.138078. 38086234

[fsn34532-bib-0048] Xu, Y. , M. Galanopoulos , E. Sismour , et al. 2020. “Effect of Enzymatic Hydrolysis Using Endo‐ and Exo‐Proteases on Secondary Structure, Functional, and Antioxidant Properties of Chickpea Protein Hydrolysates.” Journal of Food Measurement and Characterization 14, no. 1: 343–352. 10.1007/s11694-019-00296-0.

[fsn34532-bib-0049] Xu, Z. , C. Wu , D. Sun‐Waterhouse , et al. 2021. “Identification of Post‐Digestion Angiotensin‐I Converting Enzyme (ACE) Inhibitory Peptides From Soybean Protein Isolate: Their Production Conditions and In Silico Molecular Docking With ACE.” Food Chemistry 345: 128855. 10.1016/J.FOODCHEM.2020.128855. 33340899

[fsn34532-bib-0050] Yu, X. , Y. Chen , Z. Qi , Q. Chen , Y. Cao , and Q. Kong . 2023. “Preparation and Identification of a Novel Peptide With High Antioxidant Activity From Corn Gluten Meal.” Food Chemistry 424: 136389. 10.1016/J.FOODCHEM.2023.136389. 37209437

[fsn34532-bib-0051] Zeng, J. , J. Zou , J. Zhao , et al. 2023. “Chymosin Pretreatment Accelerated Papain Catalysed Hydrolysis for Decreasing Casein Antigenicity by Exposing the Cleavage Site at Tyrosine Residues.” Food Chemistry 404: 134777. 10.1016/J.FOODCHEM.2022.134777. 36444091

[fsn34532-bib-0052] Zhang, L. , Q. Xiao , Y. Wang , J. Hu , H. Xiong , and Q. Zhao . 2022. “Effects of Sequential Enzymatic Hydrolysis and Transglutaminase Crosslinking on Functional, Rheological, and Structural Properties of Whey Protein Isolate.” LWT ‐ Food Science and Technology 153: 112415. 10.1016/j.lwt.2021.112415.

[fsn34532-bib-0053] Zhang, Q. , Z. Cheng , Y. Wang , S. Zheng , Y. Wang , and L. Fu . 2021. “Combining Alcalase Hydrolysis and Transglutaminase‐Cross‐Linking Improved Bitterness and Techno‐Functional Properties of Hypoallergenic Soybean Protein Hydrolysates Through Structural Modifications.” LWT ‐ Food Science and Technology 151: 112096. 10.1016/J.LWT.2021.112096.

[fsn34532-bib-0054] Zheng, K. , Y. Wu , Q. Dai , et al. 2024. “Extraction, Identification, and Molecular Mechanisms of α‐Glucosidase Inhibitory Peptides From Defatted Antarctic Krill (*Euphausia superba*) Powder Hydrolysates.” International Journal of Biological Macromolecules 266: 131126. 10.1016/J.IJBIOMAC.2024.131126. 38527682

[fsn34532-bib-0055] Zhou, K. , S. Sun , and C. Canning . 2012. “Production and Functional Characterisation of Antioxidative Hydrolysates From Corn Protein via Enzymatic Hydrolysis and Ultrafiltration.” Food Chemistry 135, no. 3: 1192–1197. 10.1016/j.foodchem.2012.05.063. 22953842

[fsn34532-bib-0056] Zhu, B. , H. He , and T. Hou . 2019. “A Comprehensive Review of Corn Protein‐Derived Bioactive Peptides: Production, Characterization, Bioactivities, and Transport Pathways.” Comprehensive Reviews in Food Science and Food Safety 18, no. 1: 329–345. 10.1111/1541-4337.12411. 33337020

[fsn34532-bib-0057] Zhuang, M. , J. Li , A. Wang , et al. 2024. “Structurally Manipulated Antioxidant Peptides Derived From Wheat Bran: Preparation and Identification.” Food Chemistry 442: 138465. 10.1016/J.FOODCHEM.2024.138465. 38266414

